# Allelic Exchange of Pheromones and Their Receptors Reprograms Sexual Identity in *Cryptococcus neoformans*

**DOI:** 10.1371/journal.pgen.1000860

**Published:** 2010-02-26

**Authors:** Brynne C. Stanton, Steven S. Giles, Mark W. Staudt, Emilia K. Kruzel, Christina M. Hull

**Affiliations:** 1 Department of Biomolecular Chemistry, School of Medicine and Public Health, University of Wisconsin, Madison, Wisconsin, United States of America; 2 Department of Medical Microbiology and Immunology, School of Medicine and Public Health, University of Wisconsin, Madison, Wisconsin, United States of America; Washington University School of Medicine, United States of America

## Abstract

Cell type specification is a fundamental process that all cells must carry out to ensure appropriate behaviors in response to environmental stimuli. In fungi, cell identity is critical for defining “sexes” known as mating types and is controlled by components of mating type (*MAT*) loci. *MAT*–encoded genes function to define sexes via two distinct paradigms: 1) by controlling transcription of components common to both sexes, or 2) by expressing specially encoded factors (pheromones and their receptors) that differ between mating types. The human fungal pathogen *Cryptococcus neoformans* has two mating types (**a** and α) that are specified by an extremely unusual *MAT* locus. The complex architecture of this locus makes it impossible to predict which paradigm governs mating type. To identify the mechanism by which the *C. neoformans* sexes are determined, we created strains in which the pheromone and pheromone receptor from one mating type (**a**) replaced the pheromone and pheromone receptor of the other (α). We discovered that these “α**^a^**” cells effectively adopt a new mating type (that of **a** cells); they sense and respond to α factor, they elicit a mating response from α cells, and they fuse with α cells. In addition, α**^a^** cells lose the α cell type-specific response to pheromone and do not form germ tubes, instead remaining spherical like **a** cells. Finally, we discovered that exogenous expression of the diploid/dikaryon-specific transcription factor Sxi2**a** could then promote complete sexual development in crosses between α and α**^a^** strains. These data reveal that cell identity in *C. neoformans* is controlled fully by three kinds of *MAT–*encoded proteins: pheromones, pheromone receptors, and homeodomain proteins. Our findings establish the mechanisms for maintenance of distinct cell types and subsequent developmental behaviors in this unusual human fungal pathogen.

## Author Summary

All organisms that undergo sexual reproduction carry out specific mechanisms to establish different sexes. In fungi, sexual identity is typically determined by components housed within specialized regions of chromosomes known as mating-type (*MAT*) loci. *MAT*–encoded genes function to define sexes via two distinct paradigms: 1) by controlling transcription of components common to both sexes, or 2) by expressing specially encoded factors (pheromones and their receptors) that differ between mating types. The two mating types of *Cryptococcus neoformans* (**a** and α) are specified by an extremely unusual *MAT* locus. The unique architecture of this locus makes it impossible to predict which paradigm governs mating type. To identify the mechanism by which the *C. neoformans* sexes are determined, we created an α strain where the pheromones and pheromone receptor were replaced with the analogous genes from an **a** strain. We discovered that the resulting strain (α**^a^**) now behaves as if it is an **a**. It senses and responds to α cells, mates with α cells, and no longer exhibits other α-specific behaviors. Our data show that replacement of two and only two genes completely alters the sexual identity of α cells, establishing pheromones and their receptors as the determinants of sexual identity in *C. neoformans*.

## Introduction

One of the most important processes that occurs in cells is the specification of cell type, and it is by this process that cells adopt the genetic state that governs their subsequent behaviors. One mechanism of cell type-specification is the expression of genes encoded only in a given cell type. For example, X and Y chromosomes determine male and female sexes because they encode different genes, such as SRY, a Y-specific protein whose expression establishes male identity [1]. In a similar fashion, the sexes of fungi, known as “mating types,” are also determined through the expression of cell type-specific genes. The most well-characterized cell identity determination system is that of the budding yeast *Saccharomyces cerevisiae.*

In *S. cerevisiae* the two haploid cell types, **a** and α, are distinguished from one another by the actions of specific transcription factors encoded at the mating-type (*MAT*) locus [2]. *MAT* ***a*** encodes the homeodomain transcription factor **a**1, and *MATα* encodes α1 and α2, an α-domain protein and a homeodomain protein, respectively. The actions of **a**1, α1, and α2 govern control of haploid cell behavior through the differential expression of **a**-specific, and α-specific genes, including pheromone and pheromone receptor genes [3]. It is through a pheromone-pheromone receptor system that cell-cell communication occurs, and cells of opposite mating types can sense one another. Specifically, **a** cells secrete mating factor **a** pheromone (MF**a**), which binds to a receptor (Ste3) on the surface of α cells, and α cells secrete MFα pheromone, which is sensed by a receptor on the surface of **a** cells (Ste2). In response to the presence of the pheromone of a mating partner, cells undergo a cell cycle arrest and subsequent morphological changes to prepare for mating. After cell fusion, **a**1 and α2 act in concert to regulate haploid-specific genes, specifying the diploid **a**/α cell, thus completing a *MAT*-controlled regulatory circuit [2],[3].

A similar cell-cell recognition process occurs in many other fungi, including the corn smut, *Ustilago maydis*. In *U. maydis* specific pheromones and receptors are expressed in different cell types; however, in contrast to *S. cerevisiae*, these genes are not under the transcriptional control of the homeodomain proteins of the traditional *MAT* locus [4]. Instead, distinct alleles of the pheromones and their receptors are encoded within a separate *MAT* locus, and these alleles are sufficiently distinct from one another to confer cell type-specificity. In this case, haploid cells expressing distinct pheromones and receptors from the pheromone *MAT* locus sense and respond to partners of other mating types and fuse. Once compatible mating types have fused, two transcriptional regulators, bE and bW, which are encoded at the second *MAT* locus, regulate a transcriptional cascade that promotes further sexual development [4]–[6].

In a related, clinically important human pathogen, *Cryptococcus neoformans*, the determinants of haploid cell identity are unknown. *C. neoformans* contains a single *MAT* locus that is over 100 kb in size and contains 23 genes, some of which have been found to be involved in sexual development and others that appear to be essential “housekeeping” genes [7]. This locus represents an evolutionary transition from the two separate *MAT* loci found in basidiomycete fungi like *U. maydis*, to the single *MAT* locus found in ascomycetes [8]; it is unclear how components in this “fused” *MAT* locus function to specify haploid cell type. In the *C. neoformans MAT* locus, there are five genes in each mating type that represent the classic *MAT* components found in basidiomycete *MAT* loci. They include the homeodomain transcription factors *SXI2* ***a*** and *SXI1α*, the six pheromone genes *MF* ***a*** *1-3* and *MFα1-3*, and the pheromone receptors *STE3* ***a*** and *STE3α*. The transcription factors, Sxi1α and Sxi2**a**, do not appear to play any role in haploid cells, including establishment of haploid cell identity. In contrast, Sxi1α and Sxi2**a** are both necessary and sufficient to specify the dikaryotic state following cell fusion and ensure that sexual development continues [9],[10]. Conversely, the pheromones and pheromone receptors of *C. neoformans* have been shown previously to be necessary for haploid cell behaviors, such as sensing and responding to a mating partner [11]–[16]. What is not clear is whether pheromones and pheromone receptors are sufficient to confer haploid cell identity (as in *U. maydis*), or whether the actions of other regulators are required to establish the **a** and α mating types (as in *S. cerevisiae*).

To test the hypothesis that pheromones and pheromone receptors alone are sufficient to confer haploid cell identity in *C. neoformans*, we carried out a “swapping” experiment in which components from the **a** mating-type (*STE3* ***a*** and *MF* ***a*** *1*) were relocated into a strain of the opposite mating type, α (in which *STE3α* and all three copies of *MFα* had been deleted), and the effects of these modifications on mating and development were examined. The results presented here reveal that pheromones and their receptors are both necessary and sufficient to specify haploid cell identity. That is, strains that harbor a receptor and pheromone from the opposite mating type are capable of sensing and fusing with wild type cells of the same mating type. Furthermore, the fused cells are incapable of progressing through sexual development in the absence of both Sxi transcription factors; however, exogenous addition of *SXI2* ***a*** facilitates complete sexual development. These findings reveal that control of haploid cell identity in *C. neoformans* is mediated by *MAT*-encoded cell-cell communication components that are not mating type-specific in their downstream effects, and establishes a model for the maintenance of distinct cell types and developmental behaviors in this human fungal pathogen.

## Results

### Construction of modified α strains

**Figure 1:**
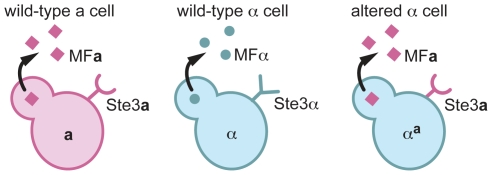
α^a^ cells are α cells that express a pheromone and the pheromone receptor from a cells. A schematic of pheromones and pheromone receptors produced by **a** cells (left), α cells (middle), and α**^a^** cells (right) is represented. **a** cells (pink) produce the Ste3**a** pheromone receptor (pink half circle/stick structure) and MF**a** pheromones (pink squares). α cells (blue) produce the Ste3α receptor (blue Y-shaped structure) and MFα pheromones (blue circles). α**^a^** cells produce only the Ste3**a** pheromone receptor and the MF**a**1 pheromone.

To test the possibility that haploid cell identity in *C. neoformans* is controlled by pheromones and pheromone receptors, a strain where the pheromone and pheromone receptor genes from one mating type were replaced with those from the opposite mating type was constructed. Specifically, we created an altered α strain by functionally replacing the *STE3α* receptor gene and the three *MFα* pheromone genes of an α cell with the *STE3* ***a*** receptor gene and three copies of the *MF* ***a*** *1* gene from an **a** cell (Figure 1).

**Figure 2:**
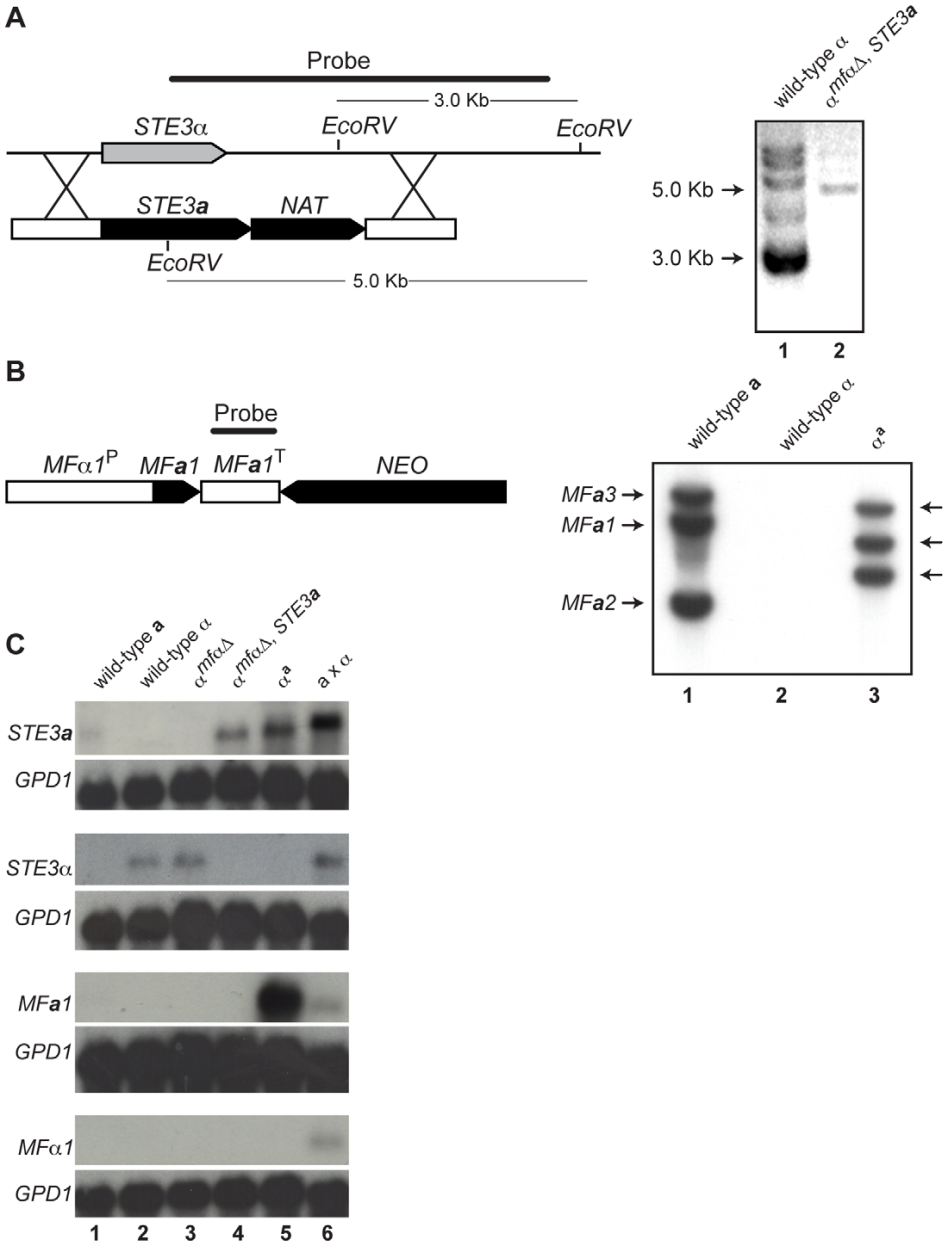
Southern and northern analyses of wild-type and modified α strains. (A) Schematic of the *STE3* ***a***/*NAT* integration event to generate the α*^mfαΔ, STE3^* ^***a***^ strain (left). Southern blot analysis confirms a single integration of the *STE3* ***a*** *-NAT* construct into the *STE3α* locus (Lane 1: ∼3 kb wild type band. Lane 2: ∼5 kb insertion band, right). (B) Schematic of the *MF* ***a*** *1*/*NEO* construct to generate the α**^a^** strain (left). Southern blot analysis reveals that 3 copies of the *MF* ***a*** *1* gene integrated into the genome. Lane 1: three wild type bands present in **a** cells. Lane 2: no *MF* ***a*** signal in α cells. Lane 3: three randomly integrated copies of *MF* ***a*** in α**^a^** cells (right). (C) Northern blot analysis of pheromone receptors (*STE3* ***a*** and *STE3α*) and pheromones (*MF* ***a*** and *MFα*). Genotypes of the test strains are indicated over the panels and probes are indicated to the left. A probe to *GPD1* was used as a loading and hybridization control. Each lane contains RNA from the following strains or crosses: Lane 1 wild type **a**, Lane 2 wild type α, Lane 3 α*^mfαΔ^*, Lane 4 α *^mfαΔ, STE3^* ^***a***^, Lane 5 α**^a^**, and Lane 6 **a** x α.

To construct the altered α strain, α cells in which all three copies of the *MFα* gene had been deleted (α *^mfαΔ^*), were transformed with a construct to replace the endogenous copy of *STE3α* with *STE3* ***a*** (Figure 2A) [13]. This replacement resulted in a complete deletion of the *STE3α* open reading frame and the precise insertion of *STE3* ***a*** under the control of the endogenous *STE3α* promoter. This strain, designated *α^mfαΔ, STE3^* ^***a***^ was confirmed by PCR (data not shown) and Southern analysis (Figure 2A) to contain a single, targeted integration of the *STE3* ***a*** gene at the *STE3α* locus. This *α^mfαΔ, STE3^* ^***a***^ strain was then transformed with a construct expressing *MF* ***a*** *1* (under the control of the *MFα1* promoter) designed to integrate randomly into the genome (Figure 2B). Multiple transformants of the resulting strain, α**^a^**, were assessed by Southern blot analysis, and several strains containing integrated copies of the *MF* ***a*** construct were carried forward in further analyses. All of the transformants confirmed to contain the *MF* ***a*** construct exhibited identical behaviors in subsequent phenotype analyses. A representative α**^a^** transformant containing three copies of the *MF* ***a*** *1* gene is shown in Figure 2B.

To assess the expression patterns of pheromones and their receptors in the constructed strains, northern blot analysis was carried out. Haploid strains were grown on V8 medium for 24 hours, and their relative transcript levels were examined (Figure 2C). The *STE3* ***a*** and *STE3α* transcripts were detected in wild type **a** and α cells, respectively, and the *STE3α* transcript was also present in the α*^mfαΔ^* strain. However, as expected, in the α*^mfαΔ^* ^, *STE3****a***^ strain where the *STE3* ***a*** *ORF* had replaced the *STE3α ORF*, only *STE3* ***a*** transcript was observed. In summary, the transcripts detected from the constructed strains were in accordance with the predicted expression patterns for each of the strains tested based on their genotypes.

While pheromone transcript was not detected in either haploid cell type (2C, bottom two panels, lanes 1 and 2), this result was not surprising because pheromone genes are transcribed at low levels in haploid cells grown on minimal medium [13]. Furthermore, pheromone transcript was not observed in strains where these genes had been deleted (α*^mfαΔ^* and α*^mfαΔ^* ^, *STE3****a***^) (lanes 3 and 4). However, when the *MF* ***a*** *1* gene was introduced into the α*^mfαΔ^* ^, *STE3****a***^ strain (resulting in the α**^a^** strain), high levels of *MF* ***a*** transcript were detected, and this high level of expression occurred in all of the *MF* ***a*** transformants (lane 5 and data not shown). Because *MF* ***a*** *1*, in this case, is under the control of the *MFα1* promoter (defined here as 1 kb of sequence upstream of the translational start site), a likely explanation for our findings is that normal repression of *MF* ***a*** *1* in haploid cells is disrupted. That is, the elements required for wild type levels of expression were likely not included in our construct, resulting in higher levels of *MF* ***a*** *1* gene expression. Because pheromones expressed from a non-native promoter do not interfere with development and the mating response, the α^a^ strain was used in our subsequent analyses [13],[14].

### Altered α strains mate with wild-type α cells

To evaluate how the series of modified α strains interacted with mating partners under sexual development conditions, crosses using various test strains were carried out and assessed microscopically for the presence of fusants after 18 hours on V8 medium. The cells used in each cross were stained with either rhodamine (Alexa Fluor 594) or fluorescein (Alexa Fluor 488) prior to mixing with partner cells so that the original mating types could be discerned after cell fusion.

**Figure 3:**
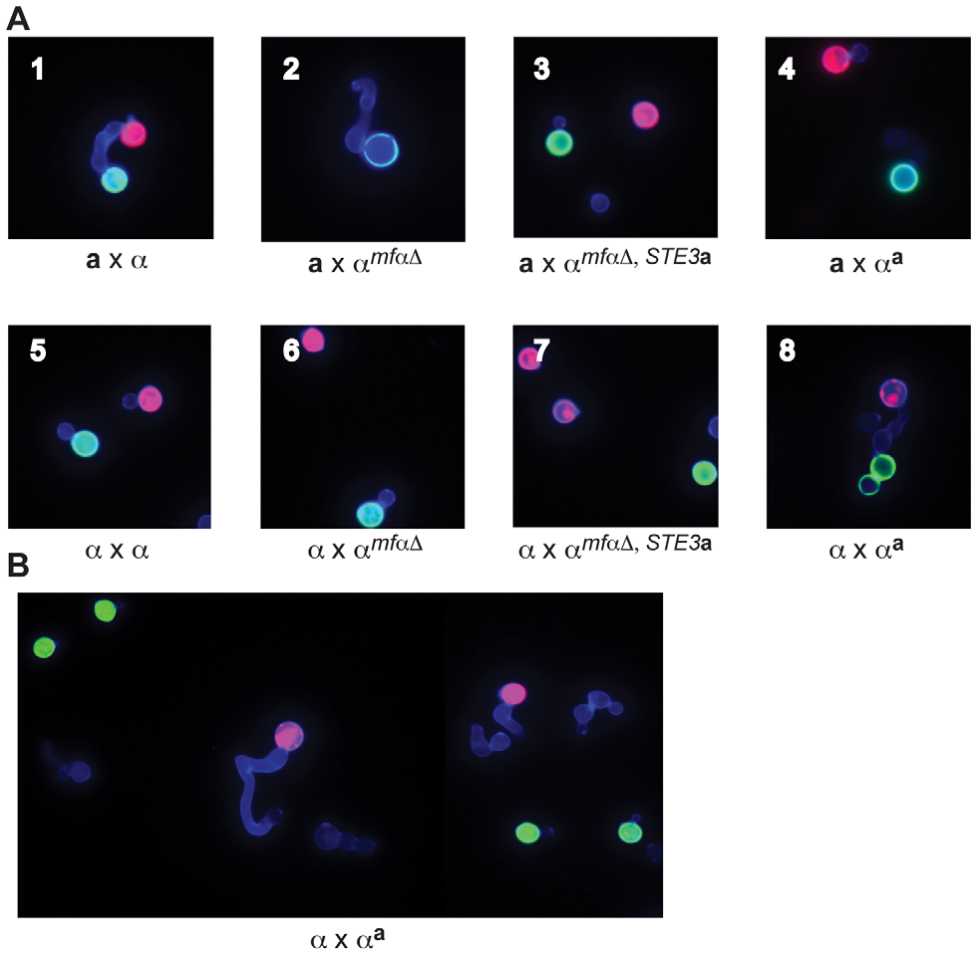
α^a^ strains fuse with wild-type α cells. (A) Sexual development assays were carried out on V8 media using differentially labeled **a** and α strains (red or green) to detect the formation of germ tubes (by α cells) and fusants (dumbbell shaped structures). A representative view from the indicated cross is displayed in each panel (1-8). In panels 1–4, **a** cells are labeled red, and α cells are labeled green. Panel 1: wild type **a** x α fusant. Panel 2: germ tube from an α*^mfαΔ^* cell; no fusants. Panels 3 and 4: no germ tubes; no fusants. In panel 5, wild type α cells are labeled red or green. In panels 6–8, wild type α cells are labeled red and modified α cells are labeled green. Panels 5–7: no germ tubes; no fusants. Panel 8: wild type α x α**^a^** fusant. (B) α**^a^** cells do not form germ tubes. Wild type α cells were labeled red, and α**^a^** cells were labeled green. Wild type α cells (red) produce germ tubes in response to **a** cells (A, panel 1) and to α**^a^** cells (A, panel 8). Similar to wild-type **a** cells, α**^a^** cells do not form germ tubes in response to α cells.

In crosses between wild type **a** and α strains, fusants were identified as “dumbbell” shaped cells, consisting of one **a** cell (red) connected to one α cell (green) via a conjugation tube (Figure 3A, panel 1). In contrast, no fusants could be identified in crosses between wild type **a** cells (red) and any modified α cells (green) (panels 2–4). Specifically, in crosses between **a** cells and α*^mfαΔ^* cells, the α*^mfαΔ^* cells responded to the presence of **a** cells by forming conjugation tubes; however, in the absence of α pheromone production, the frequency of fusion with **a** cells is predicted to be very low, and consistent with this expectation, we did not observe any fusants. Accordingly, in strains no longer harboring the *STE3α* receptor (α*^mfαΔ,STE3^* ^***a***^ and α**^a^**), the cells appear to neither form conjugation tubes (in the presence of **a** cells) nor fuse with **a** cells (panels 3 & 4). These results indicate that modified α strains, in which the *STE3α* receptor gene has been deleted, do not respond to or mate with **a** cells.

In parallel experiments, wild type α strains were crossed with the modified α strains. In a control cross, differentially labeled α cells (either red or green) were crossed and evaluated for the presence of fusants. As expected for wild type cells of the same mating type, α cells did not respond to one another or fuse (panel 5). Fusants were also not detected in crosses between wild type α cells (red) and the modified α*^mfαΔ^* and α*^mfαΔ,STE3^* ^***a***^ (green) strains (panels 6 & 7, respectively). It was not until a wild type α cell (red) was crossed with the altered α**^a^** strain (green) that fusants could be recovered at roughly wild type **a** x α levels (panel 8). Subsequent quantitative fusion assays confirmed that α x α**^a^** fusion levels are comparable to those of wild type **a** x α crosses (data not shown). This finding indicates that, as expected, pheromones and pheromone receptors are required for wild type fusion between **a** and α cells. It also reveals that the altered α**^a^** strain fuses only with wild type α cells, indicating that by simply replacing the α pheromones and receptors with those from **a** cells, cell identify was switched from α to **a**. Moreover, that change was sufficient to allow the altered α**^a^** cells to fuse with wild type α cells as though they were of opposite mating types.

In addition, the introduction of pheromone and pheromone receptor from **a** cells into α cells changes not only their ability to fuse but also the initial mating response. That is, α**^a^** strains do not produce germ tubes in response to wild type α cells, instead appearing to behave like wild type **a** cells (Figure 3B). These findings indicate that pheromones and pheromone receptors specifically control both the ability to sense a mating partner and the subsequent morphological response.

### Complete sexual development between α^a^ and wild-type α cells occurs only in the presence of both Sxi1α and Sxi2a

**Figure 4:**
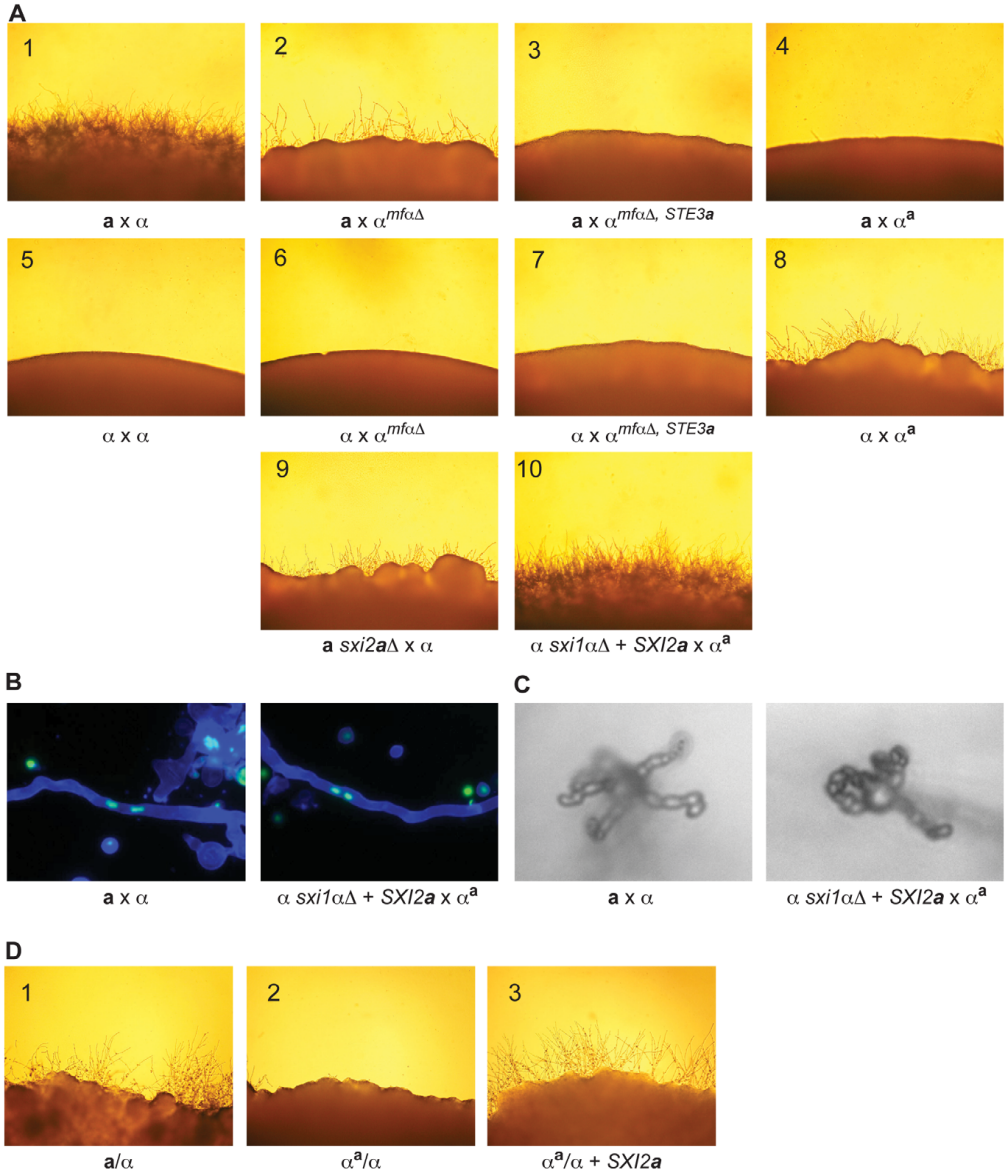
α x α^a^ crosses proceed through sexual development only in the presence of Sxi2a. (A) Sexual development assays were carried out on V8 for 72 hours, and the peripheries of crosses are shown under 200× magnification. Panels: 1) **a** x α, 2) **a** x α*^mfαΔ^*, 3) **a** x α*^mfαΔ, STE3a^*, 4) **a** x α**^a^**, 5) α x α, 6) α x α*^mfαΔ^*, 7) α x α*^mfαΔ, STE3^* ^***a***^, 8) α x α**^a^**, 9) **a** *sxi2* ***a*** *Δ* x α, 10) α *sxi1αΔ* + *SXI2* ***a*** x α**^a^**. Complete sexual development is observed only in panels 1 and 10. (B) Dikaryotic filaments are produced in the α *sxi1αΔ* + *SXI2* ***a*** x α**^a^** cross. Calcofluor stained filaments appear blue, and Sytox Green strained nuclei appear green. Both an **a** x α cross (left) and the α *sxi1αΔ* + *SXI2* ***a*** x α**^a^** cross (right) produce dikaryotic filaments (400× magnification). (C) Basidia and spores are produced in the α *sxi1αΔ* + *SXI2* ***a*** x α**^a^** cross. High resolution microscopy reveals the formation of basidia and spores from an **a** x α cross (left), and from the α *sxi1αΔ* + *SXI2* ***a*** x α**^a^** cross (right) (1000× magnification). (D) Addition of the *SXI2* ***a*** gene to α**^a^**/α diploids results in sexual development. Diploids were incubated on V8 for 72 hours, and test spot peripheries are shown under 200× magnification as follows: wild type **a**/α diploid (panel 1), α**^a^**/α diploid (panel 2), and α**^a^**/α + *SXI2* ***a*** (panel 3).

To assess the ability of the series of modified α strains to undergo sexual development, crosses were carried out and assessed for the production of filaments, basidia, and spores. In crosses between wild type **a** and α strains, florid filamentation, basidia formation, and sporulation were all visible at the periphery of the cross (Figure 4A, panel 1). However, when the modified α strains (α*^mfαΔ^*, α*^mfαΔ,STE3^* ^***a***^, and α**^a^**) were crossed with either wild type **a** or α cells, none of the combinations resulted in wild type sexual development (panels 2–8). The **a** x α*^mfαΔ^* cross (panel 2) revealed a significant reduction in sexual development, and this result is consistent with previously published data [13]. In addition, aberrant filaments were observed in the α x α**^a^** cross (panel 8). This limited, mutant filamentation was very close in appearance to crosses between strains containing deletions of *SXI2* ***a*** (panel 9). In crosses between wild type α strains and *sxi2* ***a*** *Δ* mutants, the strains fuse at wild type levels; however, only aberrant filaments are formed, and they do not progress through sexual development [9]. This phenotype occurs because both Sxi1α and Sxi2**a** must be present to specify the fused state and initiate either diploid-specific or dikaryon-specific developmental programs. Because the products of an α by α**^a^** cross contain only the Sxi1α developmental regulator (and not the Sxi2**a** regulator found only in **a** cells), such crosses would not be expected to progress through sexual development and would be expected to exhibit a *sxi2* ***a*** *Δ* cross phenotype. This is, in fact, the case. Conversely, if the Sxi proteins, pheromone, and pheromone receptors were necessary and sufficient to specify cellular identify (dikaryon vs. haploid), then simply supplying the *SXI2* ***a*** developmental regulator to an α by α**^a^** cross would result in complete sexual development.

To test the hypothesis that α x α**^a^** crosses were simply in need of *SXI2* ***a*** to continue through sexual development, we carried out crosses in which exogenous *SXI2* ***a*** was provided. Because strains containing both *SXI1α* and *SXI2* ***a*** are self-filamentous under sexual development conditions, we crossed the α**^a^** strain with an α *sxi1αΔ* strain carrying an integrated copy of *SXI2* ***a*** under the control of a constitutive promoter (*GPD1*). This cross resulted in the restoration of complete sexual development (panel 10). In addition, fluorescence and high-resolution microscopy show that dikaryotic filaments basidia, and spore chains were formed in α**^a^** x α + *SXI2* ***a*** crosses (Figures 4B, 4C). Because complete sexual development occurred between cells with nearly identical genomes (α**^a^** x α + *SXI2* ***a***), no additional information from **a** cells is required for this process.

This finding was further supported by the results of assays for diploid formation. Previous studies have shown that **a**/α diploids are self-filamentous on V8 medium, undergoing sexual development [17]. To test the ability of the α**^a^** strain to form diploids, crosses of α**^a^** by wild type **a** or α cells were carried out. α**^a^** cells fused to form mononucleate cells (presumed diploids) with wild type α cells, but not with wild type **a** cells, consistent with α**^a^** cells exhibiting **a** cell behavior. In this assay, α**^a^** cells (*ura5, NEO^R^*) were mixed with either wild type α or **a** cells (*URA5*) and incubated on V8. After 24 hours at room temperature, the co-cultures were plated under double selection (*Ura^−^* + G418) at 37°C to select for mononucleate diploids [17]. No colonies grew from the **a** x α**^a^** crosses, indicating that no **a**/α**^a^** diploids were formed. In contrast, abundant colonies were recovered from the α x α**^a^** crosses, indicating that α**^a^**/α diploids were formed. The resulting α**^a^**/α strains were determined to be mononucleate (data not shown) and were evaluated for the ability to undergo sexual development by incubation on V8 at room temperature (Figure 4D). As in α x α**^a^** crosses, sexual development was not observed (panel 2); however, the addition of the *SXI2* ***a*** gene to the α**^a^**/α strain resulted in full sexual development on V8, similar to that of a wild type **a/**α diploid (panels 3 and 1, respectively). Taken together, these results demonstrate that cell identity and sexual development are controlled entirely by three kinds of genes in *C. neoformans*: pheromones, pheromone receptors, and homeodomain proteins.

### The α^a^ strain undergoes α fruiting

**Figure 5:**
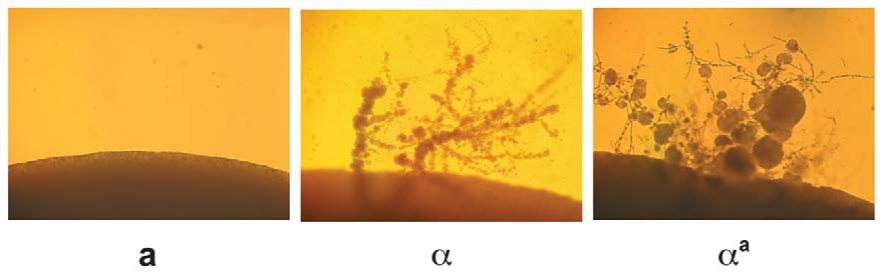
α^a^ cells undergo α fruiting. Fruiting assays were carried out on filament agar for 14 days at room temperature in the dark. The peripheries of colonies are shown at 100× magnification. Left panel: wild type **a** cells. Middle panel: wild type α cells. Right panel: α**^a^** cells.

Monokaryotic or α fruiting is another form of sexual development that takes place in *C. neoformans*; however, this process is specific to α cells, which form filaments, basidia, and spores under severe nutrient limitation and desiccation [10],[18],[19]. Although fruiting is known to be an α specific process, the factors responsible for α fruiting are unknown. To assess whether changing haploid cell identity influences α fruiting, fruiting assays were carried out with wild type **a**, wild type α, and α**^a^** strains. Strains were incubated on filament agar for 14 days at room temperature in the dark and evaluated for the production of filaments and spores. We observed that the α**^a^** strain undergoes α fruiting that is indistinguishable from wild type α cells (Figure 5), indicating that this α-specific process does not require α-specific pheromones or pheromone receptor, and is not influenced by the presence of **a**-specific pheromones and pheromone receptor.

## Discussion

Sexual development is an essential part of the life cycle for most eukaryotic organisms and requires mechanisms to ensure that development takes place between compatible partners. In both ascomycete and basidiomycete fungi, mating type is specified by components of the *MAT* locus [20]; however, the mechanisms by which *MAT* components act to confer cell identity vary widely among fungi [21],[22]. In this study we discovered that the basidiomycete *C. neoformans* establishes haploid cell type through the expression of mating type-specific pheromones and pheromone receptors. By replacing the pheromone and the pheromone receptor genes of α cells with those from **a** cells, we created an α cell with the haploid identity of an **a**. This altered strain exhibited the mating behaviors of an **a** cell, including the ability to fuse with an α mating partner. This result shows that no additional mating type-specific factors are necessary to denote haploid cell type. Furthermore, the addition of the **a**-specific factor *SXI2* ***a*** was then sufficient to facilitate filamentation and spore formation. Through these studies we have identified the factors that determine haploid cell identity (pheromones and their receptors), thus answering the question of how the *MAT* locus of *C. neoformans* controls cell type-specification.

### Cell identity in fungi

There is a long history of investigating pheromone/pheromone receptor function in fungi, particularly in *S. cerevisiae*, and myriad experiments have been carried out to understand the roles of two phylogenetically unrelated receptors *STE2* (α factor receptor) and *STE3* (**a** factor receptor). Through altered expression experiments it was found that mating type is controlled by the identity of the expressed surface receptor. Experiments where the receptor expression patterns were reversed in **a** and α cells led to changes in mating behaviors. For example, α cells that express only *STE2* (and not *STE3*), can sense and respond to α pheromone and therefore mate as **a** cells and vice versa. Thus, in *S. cerevisiae*, both **a** and α cells contain the *STE2* and *STE3* genes (and both pheromone genes), and it is the actions of transcription factors that impart differential expression patterns that determine cell identity [23],[24].

This is in sharp contrast to other fungi, like *U. maydis*, in which different cell types encode distinct pheromones and pheromone receptors. Experiments in *U. maydis* where the pheromones and pheromone receptors have been “swapped” between mating types demonstrate that mis-expression of pheromones and pheromone receptors is sufficient to alter cell identity. In this case, the pheromone receptors have distinct binding specificities but have descended from a common ancestor [4]. This is also the case for other basidiomycetes, including *S. commune* and *C. cinerea*. Within these fungi, different mating types encode distinct alleles of pheromones and pheromone receptors; pheromone and pheromone receptors do not govern mating, and cell fusion occurs independently of pheromone signaling. Instead, pheromones and pheromone receptors control post-fusion sexual development [25]–[27]. These examples speak to the wide variety of strategies employed by pheromones and pheromone receptors to control sexual development in fungi and emphasize the need to investigate pheromone/pheromone receptor function on a case-by-case basis.

### *C. neoformans* pheromones and pheromone receptors

In *C. neoformans*, both **a** and α cells contain mating type-specific pheromone genes (*MF* ***a*** *1*, *2*, and *3* in **a** cells, and *MFα1*, *2*, and *3* in α cells), but the regulation of these genes differs between mating types. In **a** cells, *MF* ***a*** pheromone is highly induced on V8 agar (or under conditions of nutrient limitation) in the absence of a mating partner. In addition to induction under nutrient limiting conditions, *MFα* pheromone expression is also activated by factors secreted by **a** cells (MF**a** pheromone) [13], and expression of the *MFα* genes is dramatically upregulated upon exposure to synthetic MF**a** pheromone [12],[13].

In the experiments carried out here, expression of the *MF* ***a*** *1* gene in α**^a^** cells is driven by the *MFα1* promoter. The *MF* ***a*** *1* construct was originally engineered in this fashion to avoid any cell type-specific regulation that might be imposed upon an **a**-specific promoter in an α cell. However, in α**^a^** strains where *MF* ***a*** *1* gene expression was driven by the *MF* ***a*** *1* promoter, identical phenotypes and expression patterns were observed.

Attempts to test the roles of the pheromone receptors were not as straight forward, and initially, the *STE3* ***a*** cassette was transformed into a wild type α strain to replace the *STE3α* gene at its native locus. However, after multiple attempts, no transformants were recovered (Ekena, Giles, and Hull, unpublished data). Transformants were recovered only from strains in which the pheromone genes were deleted previously. These findings, while far from conclusive, intimate that cells expressing both *STE3* ***a*** and α pheromone may engage in autocrine signaling that affects cell growth.

A similar observation has been made in *S. cerevisiae*, in which a strain that expresses both a pheromone and its cognate receptor undergoes G1 cell cycle arrest [24]. In *S. cerevisiae* this arrest is transient; however, if a similar arrest is occurring in *C. neoformans*, the cells do not appear to recover. This is in contrast to *U. maydis* where cells that have been engineered to express both receptor alleles do not arrest, are fully viable, and are mating competent. It remains to be determined if and how autocrine signaling may be functioning in *C. neoformans*.

### Receptor identity influences mating morphology but not fruiting morphology

In *C. neoformans*, **a** and α cells carry out distinct functions to initiate the mating process. For example, in response to nitrogen limitation, **a** cells secrete MF**a** pheromone, and α cells respond to the presence of the MF**a** pheromone by formation of germ tubes. This morphological response is specific to α cells, and only these cells (and not **a** cells) form germ tubes as an initial mating response [11],[28],[29]. Interestingly, α**^a^** cells not only recognize and fuse with α cells (as would be expected for an **a** cell), but they also exhibit the morphology of an **a** cell, and do not produce germ tubes. This finding indicates that the morphological response to pheromone is mediated by the pheromone receptor (not downstream signaling events) and is independent of other mating type-specific factors. Therefore, pheromones and pheromone receptors alone dictate both the detection of a mating partner and the initial morphological response of haploid cells in *C. neoformans*. Interestingly, however, changes in mating identity did not influence the α-specific process of α fruiting.

Monokaryotic (or α) fruiting is a process that is specific to α cells and was recently shown to be a form of sexual development [19]. While the mechanisms underlying α fruiting are relatively undefined, it is well established that the α fruiting pathway is independent of Sxi1α and Sxi2**a**, and that this process is attenuated in strains in which *STE3α* or the *MFα* genes have been deleted [12],[13],[19]. Our data reveal that although α fruiting is primarily an α-specific process, it occurs regardless of whether pheromones and pheromone receptor are derived from **a** or α cells.

In summary, while the α fruiting process occurs despite the identity of pheromones and their receptors, these factors are wholly responsible for determining the initial mating response and haploid cell identity. The findings presented here establish a complete cell identity determination profile for *C. neoformans* and enhance our understanding of the myriad strategies by which fungi specify mating types and undergo sexual development.

## Materials and Methods

### Constructing modified α strains

To generate the α*^mfαΔ, STE3^* ^***a***^ strain (CHY1900), the α*^mfαΔ^* (WSC17) strain was biolistically transformed with a fragment containing *STE3* ***a*** and a nourseothricin resistance (*NAT^R^*) cassette that was flanked by ∼1 kb of sequence from upstream and downstream of the *STE3α* open reading frame (ORF) [13]. Nourseothricin resistant transformants were screened by PCR for proper integration of the deletion/insertion construct into the *STE3α* locus, and putative deletion/insertion strains were confirmed by Southern blot analysis [30]. To generate the α**^a^** strain (CHY1901), the α*^mfαΔ, STE3^* ^***a***^ strain was biolistically transformed with a randomly integrating fragment of DNA containing *MF* ***a*** *1* (under the control of the *MFα1* promoter) and a neomycin resistance (*NEO^R^*) cassette. Neomycin resistant transformants were screened for copy number of the insertion using Southern blot analysis [30]. The α**^a^**/α strain (CHY2049) was generated by crossing the 5-FOA resistant α**^a^** strain (CHY1517) by wild type α cells (JEC21) and selecting for fusants at 37°C on minimal medium containing neomycin. To generate the α**^a^**/α*^SXI2^* ^***a***^ strain (CHY2096), 5-FOA resistant α**^a^**/α cells were biolistically transformed with a randomly integrating fragment of DNA containing the *SXI2* ***a*** (under the control of the *GPD1* promoter) and *URA5* genes.

### Northern blot analysis

RNA was prepared from *C. neoformans* cells using a hot phenol extraction [30]. Strains were grown on solid V8 medium for 24 hours at room temperature. Northern blots were carried out according to standard protocols with 10 µg of total RNA used for each sample [30]. The glycerol-3-phosphate dehydrogenase gene (*GPD1*) probe was PCR-generated using CHO651 (CGTCGTTGAATCTACCGGTG) & CHO652 (CACCAGCAATGTAAGAGATG). All other probes were generated by PCR using the following oligonucleotides: CHO2030 (CCCCGACTATCCCTTTTGGAATCTCACTGC) & CHO2031 (GGCGAACAGTTCTTCGGGATATTGTGATACC) for *STE3* ***a***, CHO2032 (GCACGACCTCAGCCTCGTCATTTTCAGCGG) & CHO2033 (CCGTATCCAGCAGCAATGATCGTCAGC) for *STE3α*, CHO2052 (GACTTACTCTTGCGTTATTGCTTAAAGTGGG) & CHO1917 (GAAAAGAGGTACGAGTAGAT) for *MF* ***a*** *1*, and CHO2053 (TTGTGTCATCGCCTAGACCCAACGTCCCC) & CHO2054 (CCATCTAAACAAGTCCCATACGCTTCGTTACC) for *MFα1*. Radiolabeled probes (Decaprime II kit; Ambion) were used in hybridization reactions as described previously [31]. Hybridizations and washes were carried out at 65°C as described previously [30].

### Strain manipulations and media

All strains used were of the serotype D background (Table 1) and were handled using standard techniques and media as described previously [32],[33]. Sexual development assays were conducted on 5% V8 medium at room temperature in the dark for 2–4 days and were evaluated by observing the periphery of test spots. The mating tester strains used were JEC20 (**a**) and JEC21 (α), and crosses were photographed at 100X magnification [18]. Spores were photographed at 400X magnification. Fruiting assays were carried out by growing cells on filament agar (0.67% yeast nitrogen base (without amino acids or ammonium sulfate) containing 100 nM (NH_4_)_2_SO_4_ at room temperature in the dark for 14 days. The periphery of test spots were photographed at 100X magnification.

**Table 1:** Strains.

Number	Name	Genotype	Strain origin
JEC20	**A**	wild type **a**	Wickes et al. 1990
JEC21	α	wild type α	Wickes et al. 1990
WSC17	α*^mfαΔ^*	α *mfα1Δ*::*ADE2 mfα2,3Δ*::*URA5*	Shen et al. 2000
CHY769	**a** *^sxi2^* ^***a****Δ*^	**a** *sxi2* ***a*** *Δ::URA5*	Hull et al. 2005
CHY786	**a**/α	*lys1/LYS1 ade2/ADE2 lys2/LYS2*	This study
CHY1517	α**^a^** ^ 5-FOA^	α *mfα1Δ*::*ADE2 mfα2,3Δ*::*URA5,* 5-FOA^R^ *ste3αΔ*::*STE3* ***a*** *-NAT^R^ MF* ***a*** *1-NEO^R^*	This study
CHY1696	α*^sxi1αΔ, SXI2^* ^***a***^	α *sxi1αΔ::NAT^R^ + GPD-SXI2* ***a*** *-URA5*	This study
CHY1900	α*^mfαΔ, STE3^* ^***a***^	α *mfα1Δ*::*ADE2 mfα2,3Δ*::*URA5 ste3αΔ*::*STE3* ***a*** *-NAT^R^*	This study
CHY1901	α**^a^**	α *mfα1Δ*::*ADE2 mfα2,3Δ*::*URA5 ste3αΔ*::*STE3* ***a*** *-NAT^R^ MF* ***a*** *1-NEO^R^*	This study
CHY2049	α**^a^**/α	α**^a^**/α *mfα1Δ*::*ADE2*/*MFα1 mfα2*,*3Δ*::*URA5,* 5-FOA^R^ */MFα2*,*3 ste3αΔ*::*STE3* ***a*** *-NAT^R^*/*STE3α MF* ***a*** *1-NEO^R^*	This study
CHY2096	α**^a^**/α*^SXI2^* ^***a***^	α**^a^**/α *mfα1Δ*::*ADE2*/*MFα1 mfα2*,*3Δ*::*URA5,* 5-FOA^R^/*MFα2*,*3 ste3αΔ*::*STE3* ***a*** *-NAT^R^*/*STE3α MF* ***a*** *1-NEO^R^* p*GPD1*-*SXI2* ***a*** *-URA5*	This study

### Microscopy and staining

Fluorescent microscopy was carried out on a Zeiss Axioskop 2 fluorescent microscope fitted with an Axiocam MRM REV3 digital camera and corresponding AV4 software. Light microscopy was carried out using a Zeiss Axioplan microscope fitted with a 10X long working distance objective. Photographs were taken with a Nikon Coolpix 5400 camera mounted on the microscope. To label fusants, **a** and α cells were grown to stationary phase in yeast extract peptone dextrose (YPD) liquid medium and were suspended in 1X phosphate buffered saline (PBS). Carboxylic acid, succinimidyl-ester Alexa Fluor 488 or 594 dyes (Invitrogen, Carlsbad CA) were coupled to **a** or α yeast cells in 100 mM potassium phosphate buffer pH 7.5 for 30 min at room temperature. After washing, differentially labeled cells were mixed in 1X PBS and spotted onto V8 agar. Spots were incubated at room temperature for 6 hours and were then resuspended into a mounting solution of 1X PBS containing 0.4 µg/ml calcofluor white MR2 (Sigma-Aldrich). For filament staining, crosses were incubated on a thin layer of V8 media on a microscope slide and incubated at room temperature for 24 hours. Cells were fixed with 2.5% gluteraldehyde/4% paraformaldehyde for 30 minutes. Cells were washed twice with 1X PBS containing 0.1% Triton X-100, once with 1X PBS, and incubated in 1X PBS containing 0.4 µg/mL calcofluor white to stain septa, and 1nM Sytox green (Invitrogen) to stain nuclei for 20 minutes. Cells were washed in 5% glycerol with 20% DABCO antifade (Sigma) prior to visualization.
